# Transcriptome analysis of *Citrus limon* infected with *Citrus yellow* *vein clearing virus*

**DOI:** 10.1186/s12864-023-09151-5

**Published:** 2023-02-07

**Authors:** Yu Bin, Qi Zhang, Yue Su, Chunqing Wang, Qiqi Jiang, Zhen Song, Changyong Zhou

**Affiliations:** Citrus Research Institute, Southwest University, Beibei, Chongqing, 400712 China

**Keywords:** CYVCV, RNA-Seq, Phytohormone, Photosynthesis, Symptom development

## Abstract

**Background:**

*Citrus yellow vein clearing virus* (CYVCV) is the causative agent of citrus yellow vein clearing disease, and poses a serious threat to the lemon industry in Asia. The common symptoms of CYVCV-infected lemon plants are leaf crinkling, leaf chlorotic mottling, and yellow vein clearing. However, the molecular mechanisms underlying CYVCV-citrus interaction that responsible for symptom occurrence is still unclarified. In this study, RNA-seq was performed to analyze the gene expression patterns of ‘Eureka’ lemon (*Citrus limon* Burm. f.) plants in response to CYVCV infection.

**Results:**

There were 3691 differentially expressed genes (DEGs) identified by comparison between mock and CYVCV-infected lemon plants through RNA-seq. Bioinformatics analyses revealed that these DEGs were components of different pathways involved in phenylpropanoid biosynthesis, brassinosteroid biosynthesis, flavonoid biosynthesis and photosynthesis. Among these, the DEGs related to phytohormone metabolism and photosynthesis pathways were further enriched and analyzed. This study showed that different phytohormone-related genes had different responses toward CYVCV infection, however almost all of the photosynthesis-related DEGs were down-regulated in the CYVCV-infected lemon plants. The obtained RNA-seq data were validated by RT-qPCR using 12 randomly chosen genes, and the results of mRNA expression analysis were consistent with those of RNA-seq.

**Conclusions:**

The phytohormone biosynthesis, signaling and photosynthesis-related genes of lemon plants were probably involved in systemic infection and symptom occurrence of CYVCV. Notably, CYVCV infection had regulatory effects on the biosynthesis and signaling of phytohormone, which likely improve systemic infection of CYVCV. Additionally, CYVCV infection could cause structural changes in chloroplast and inhibition of photosynthesis pathway, which probably contribute to the appearance of leaf chlorotic mottling and yellow vein clearing in CYVCV-infected lemon plants. This study illustrates the dynamic nature of the citrus-CYVCV interaction at the transcriptome level and provides new insights into the molecular mechanism underlying the pathogenesis of CYVCV in lemon plants.

**Supplementary Information:**

The online version contains supplementary material available at 10.1186/s12864-023-09151-5.

## Background

Citrus yellow vein clearing disease (CYVCD) causes serious yield losses in lemon production in Pakistan, Turkey, India, Iran, and China [1–4]. Nowadays, CYVCD is widely distributed in major citrus growing regions in China, and is recognized as the most severe disease that affects lemon production [5]. CYVCD is caused by the *citrus yellow vein clearing virus* (CYVCV), which is transmitted between citrus plants mainly via citrus whitefly (*Dialeurodes citri* Ashmead), contaminated knives, and pruning blades [3, 6–9]. CYVCV is usually asymptomatic in most citrus species, cultivars and hybrids, but severe leaf distortion and yellow vein clearing are found in sour orange and lemon [5]. CYVCV belongs to the genus *Mandarivirus* in the family *Alphaflexiviridae,* and its genome contains a positive-sense single-stranded RNA [3]. The genome of CYVCV (~ 7.5 kb) encodes six proteins: a putative viral replication-associated polyprotein-replicase (REP), the triple gene block proteins (TGBp1, TGBp2 and TGBp3), a putative capsid protein (CP) and a hypothetical nucleic-acid-binding protein (NaBP) [3, 10]. CP has been identified to be a strong RNA silencing suppressor (RSS) [11]. However, the molecular mechanisms underlying CYVCV-citrus interaction that is responsible for symptom occurrence is still unclarified.

Phytohormones play a vital role in tuning abiotic and biotic responses in plants. They are involved in a variety of complex networks, through which they modulate responses to different stimuli [12]. The phytohormones include salicylic acid (SA), jasmonic acid (JA), ethylene (Et), abscisic acid (ABA), auxins (AUXs), gibberellins (GAs), cytokinins (CKs), and brassinosteroids (BRs) [13]. These eight signaling molecules are shown to play important roles in mediating plant disease resistance [14, 15]. Phytohormone regulatory systems are also involved in immune response to a viral infection [12]. The complex interaction between viruses and their host plants often leads to system development, including stunted growth, leaf mottling and leaf yellowing [16]. To a certain extent, the symptoms induced by viral infection may be ascribed to the changes in the amount of a particular phytohormones and can be mimicked by the removal or application of these phytohormones [17]. Hormones have synergistic or antagonistic interrelations, and some hormones can prevail over others under certain conditions [12].

Chloroplast is a common target of plant viruses for viral propagation or pathogenesis. However, chloroplast and its components play a key role in plant defense against viruses. Chloroplast photosynthesis-related proteins/genes have been reported to play an essential role during the chloroplast-virus interaction [18, 19]. Some viruses can affect the host’s photosynthesis, leading to chlorotic leaves and severe mosaic symptoms [20, 21]. Previous research has shown that several proteins involved in the Benson-Calvin cycle and photosynthetic electron-transport chain are down-regulated in *Nicotiana benthamiana* plants during pepper mild mottle virus (PMMoV) infection [22]. Moreover, alteration of photosynthesis is an important strategy for pathogenesis employed by viruses to facilitate infection [18, 19]. The disruption of normal chloroplast constituents and functions may be responsible for the development of chlorosis symptoms related to viral infection [23].

Recently, high-throughput RNA sequencing (RNA-seq) has been utilized to comprehensively analyze the expression patterns of host genes during virus-plant interaction. In addition, transcriptomics analysis has also been conducted to determine the specific metabolic changes related to symptom development, which resemble both physiological and pathological changes in the host plants. Elucidating the responses of lemon to CYVCV infection is crucial for uncovering the mechanisms of lemon resistance to CYVCV infection, as well as the pathophysiology and molecular mechanisms underlying symptom development. In this research, the transcriptomic profiles of lemon plants infected with CYVCV were analyzed by RNA-seq. The findings provide novel insights into the molecular mechanism of CYVCV pathogenesis during virus-plant interaction. To our knowledge, this study is the first to identify the transcriptomic changes in lemon plants after CYVCV infection.

## Results

### Transcriptomic analysis of RNA-seq data

**Fig. 1:**
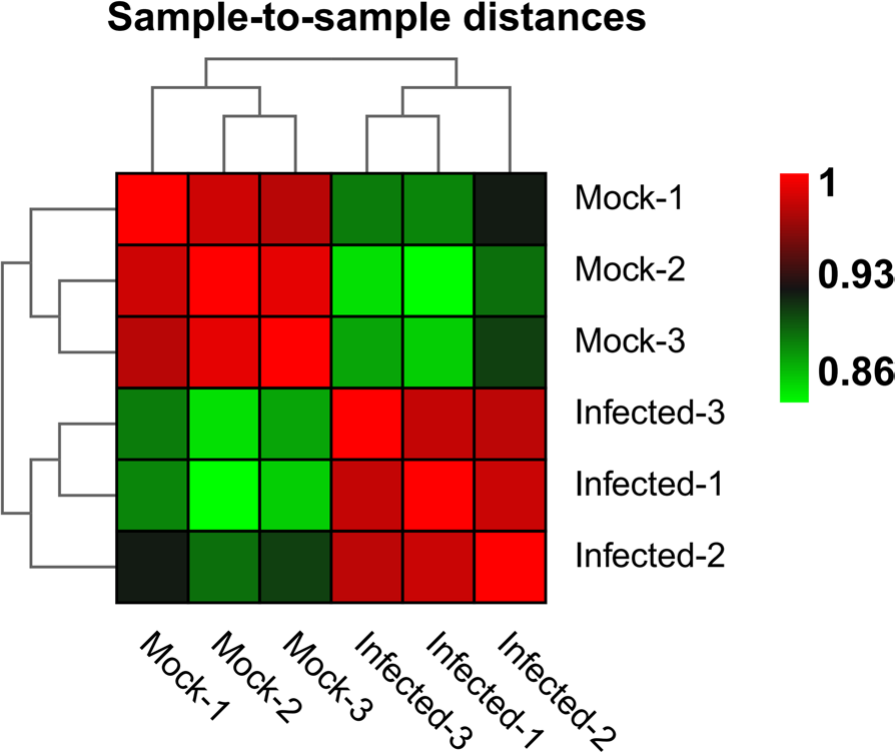
Sample to sample clustering analysis for examining batch effects and their similarity. Mock represents mock-inoculated plants, while Infected represents CYVCV-infected plants

**Fig. 2:**
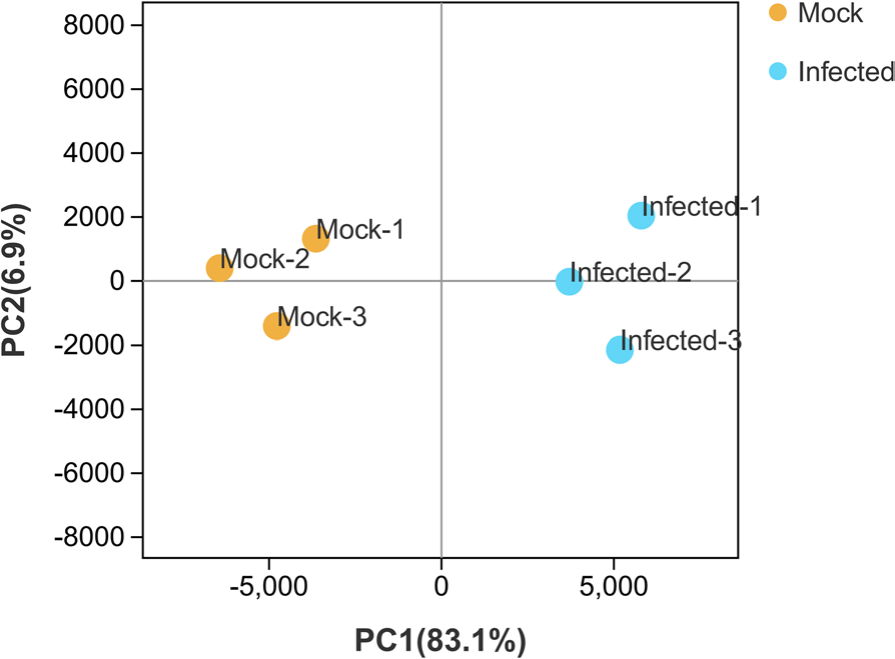
Principal component analysis (PCA) for classifying gene expression patterns. The first and second PCAs explained PC1 (83.1%) and PC2 (0.47%) of the variances, respectively. Mock represents mock-inoculated plants, while Infected represents CYVCV-infected plants

To obtain a global overview of the transcriptomic changes in ‘Eureka’ lemon (*Citrus limon* Burm. f.) infected with CYVCV, the expression patterns of CYVCV-infected lemon (Infected-1, Infected-2 and Infected-3) were compared to those of mock-inoculated lemon (Mock-1, Mock-2 and Mock-3) via high-throughput RNA-seq. In total, 55,539,014 to 65,060,414 and 53,883,178 to 62,815,094 raw reads were detected for Infected group (Infected-1, Infected-2 and Infected-3) and Mock group (Mock-1, Mock-2 and Mock-3), respectively. After removing the adapter sequences and low-quality reads, the clean reads ranged from 54,821,468 to 64,308,378 for the Infected group and 53,294,352 to 61,867,500 for the Mock group (Table 1). Sample to sample clustering analysis revealed that the expression level of DEGs between three replicates was reproducible and the batch effect was controlled (Fig. 1). Principal component analysis (PCA) was conducted for both mock and CYVCV infection groups. The mock group was located around the junction of the second and third quadrants, while the CYVCV infection group was located around the junction of the first and fourth quadrants, suggesting a good reproducibility for the biological replicates within the same group but different between the two groups (Fig. 2).

**Table 1:** Summary of mapping reads of the RNA-seq

Statistics term	Mock-1	Mock-2	Mock-3	Infected-1	Infected-2	Infected-3
Raw reads	62,815,094	53,883,178	55,416,112	55,539,014	57,813,818	65,060,414
^Clean reads^	61,867,500	53,294,352	54,780,098	54,821,468	57,163,606	64,308,378
UnMapped	16,562,604	14,080,622	15,226,717	14,906,716	15,290,728	16,990,164
Mapped reads	45,301,896	39,213,730	39,553,381	39,914,752	41,872,878	47,318,214
Mapping rate	72.68%	71.83%	71.87%	72.34%	72.88%	73.21%
Unique mapping	42,987,758	35,056,240	37,981,251	38,014,332	40,124,646	45,314,666
Unique mapping rate	70.91%	70.13%	70.16%	70.53%	71.17%	71.44%

The clean reads were then mapped to the reference genome of *Citrus sinensis*. (http://www.ncbi.nlm.nih.gov/genome/10702)

### Identification of differentially expressed genes (DEGs) in lemon plants infected with CYVCV

**Fig. 3:**
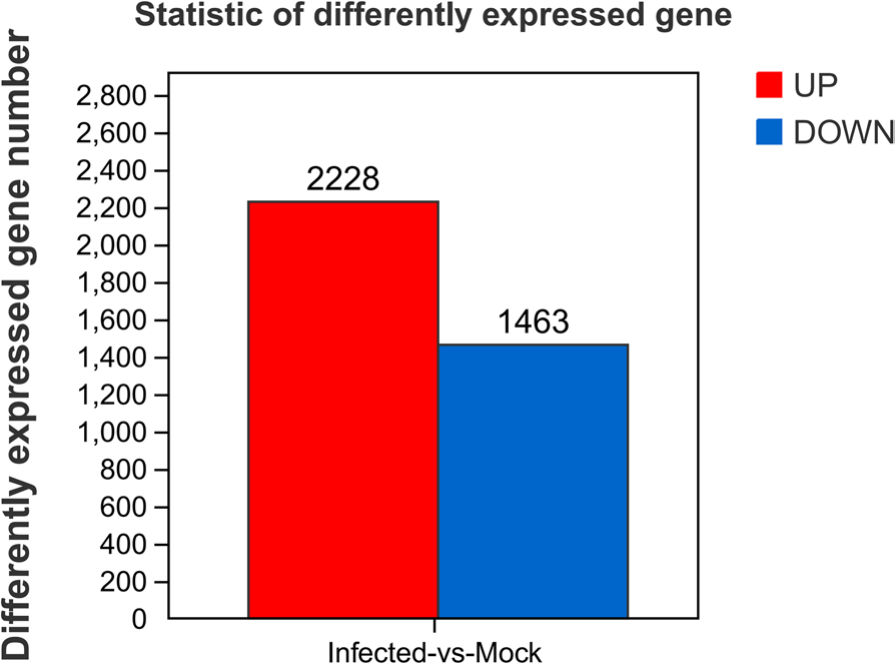
Significant differentially expressed genes (DEGs) in mock vs. CYVCV-infected libraries. Down- or up-regulated DEGs in response to CYVCV infection. CK represents mock-inoculated plants, while Infected represents CYVCV-infected plants

**Fig. 4:**
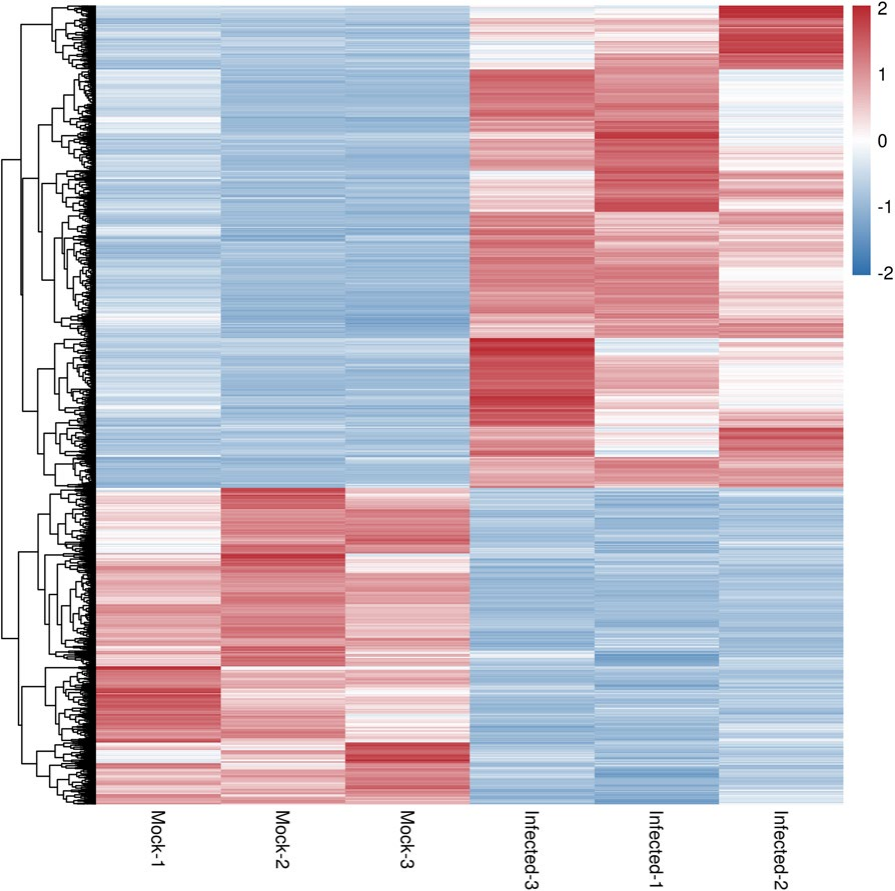
Hierarchical clustering heatmap of DEGs after CYVCV infection. Each row indicates DEG expression, while each column indicates a library. The colors red, white and blue denote high, medium and low expression patterns of DEGs, respectively. Mock represents the mock-inoculated plants, Infected represents CYVCV-infected plants

DESeq2 software was used to identify DEGs between the mock and CYVCV infection groups. A total of 1,463 down-regulated and 2,228 up-regulated genes were identified (Fig. 3; Table S2). To determine the transcriptional changes in response to CYVCV infection, hierarchical clustering analysis was performed to reveal the expression patterns of DEGs. The results demonstrated that the expression levels of mock and CYVCV-infected plants were different from each other, but were similar in the replicates of the same group. The number of up-regulated DEGs was markedly higher than that of down-regulated DEGs in lemon plants after CYVCV infection (Fig. 3 and Fig. 4).

### Functional analysis of DEGs

**Fig. 5:**
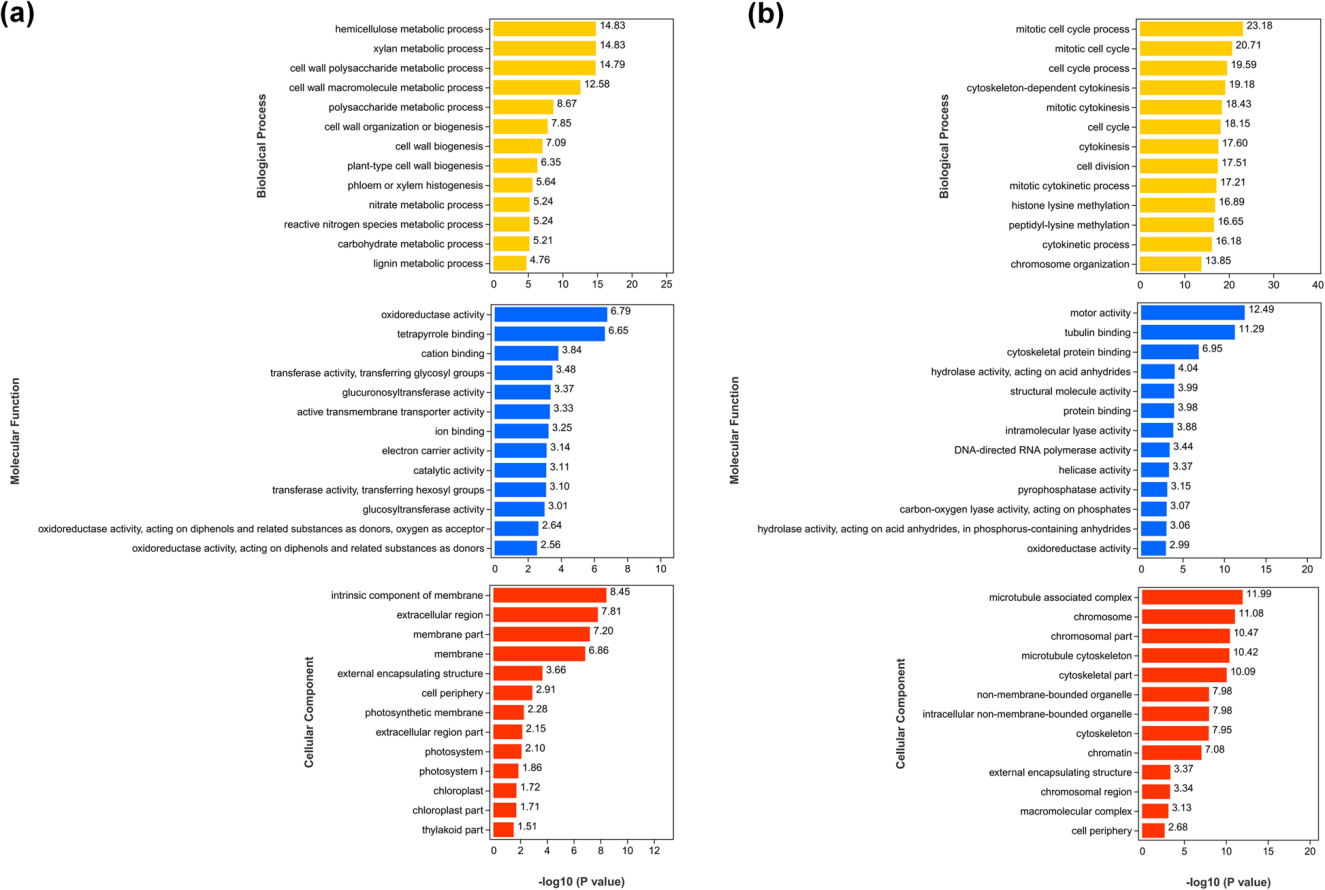
Gene ontology (GO) enrichment analysis of DEGs after CYVCV infection. **a, b** Top 13 GO terms significantly enriched for down-regulated and up-regulated DEGs. A term with *p*-value ≤ 0.05 was deemed significant and enriched GO term was determined with hypergeometric test. The y-axis indicates the significantly enriched GO term, and the -log10 (*P*-value) was used to depict the significance of GO term for each DEG. Different colors are employed to differentiate molecular functions, cellular components, and biological processes

Gene ontology (GO) enrichment analysis was conducted to elucidate the functions of DEGs. Both up- and down-regulated DEGs can be classified under biological process, cellular component and molecular function (Fig. 5; Table S3). The top 13 significantly enriched GO terms for up- and down-regulated DEGs (*p*-value ≤ 0.05) are displayed in Fig. 5. For up-regulated DEGs, the most significantly enriched GO terms were ‘mitotic cell cycle process’, ‘motor activity’ and ‘microtubule associated complex’, while for down-regulated DEGs, ‘hemicellulose metabolic process’, ‘oxidoreductase activity’ and ‘intrinsic component of membrane’ were the most significantly enriched terms (Fig. 5; Table S3). Moreover, we found that in the cellular component category for down-regulated DEGs, DEGs were mainly associated with ‘membrane part’, ‘photosynthetic membrane’, ‘photosystem’, ‘chloroplast’ and ‘thylakoid part’ (Fig. 5; Table S3).

### Pathway analysis of DEGs

**Fig. 6:**
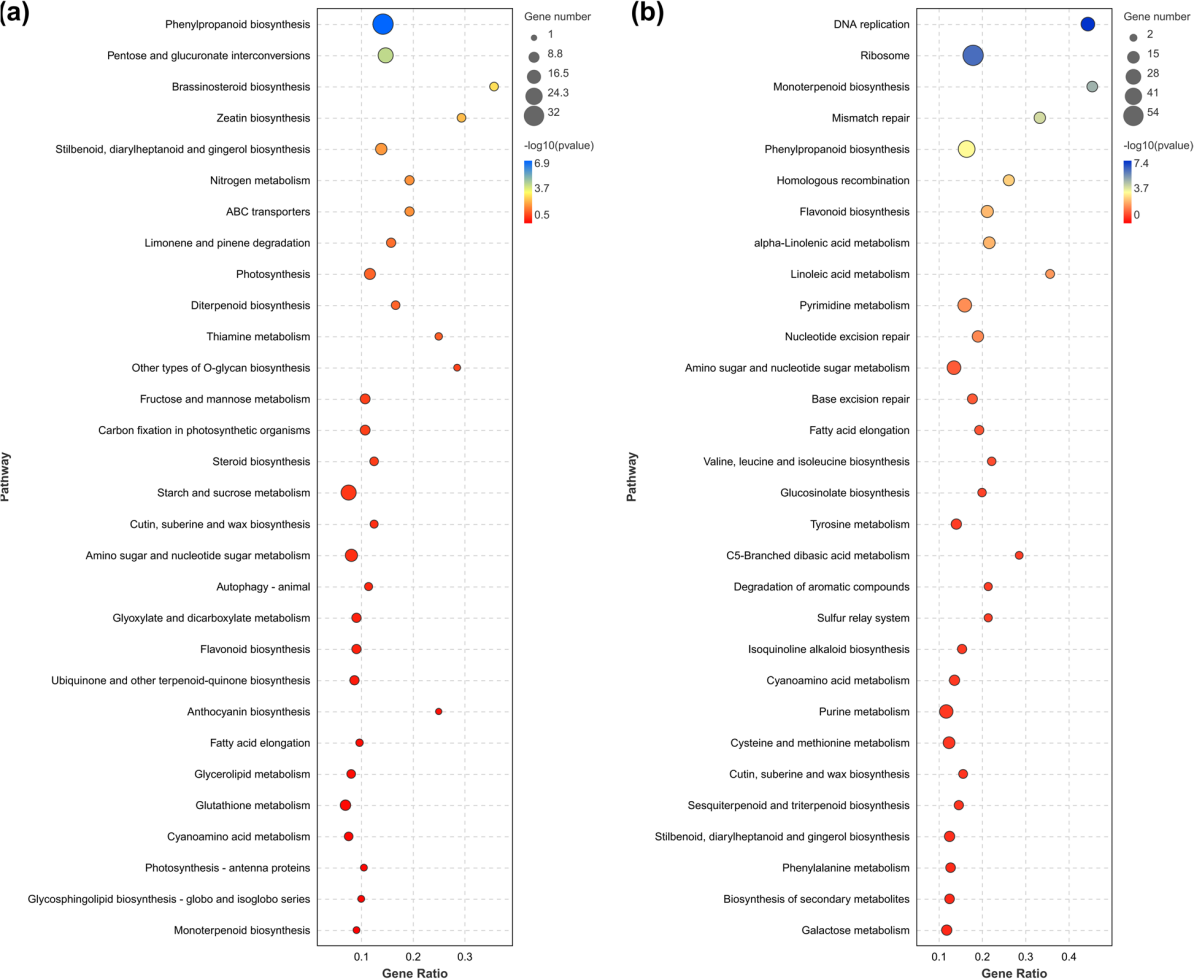
Kyoto encyclopedia of genes and genomes (KEGG) pathway analysis of DEGs [73–75]. Top 30 KEGG terms enriched for down-regulated (**a**) and up-regulated DEGs (**b**). The size of each circle indicates the number of DEGs enriched in the corresponding term. A term with *p*-value ≤ 0.05 was deemed significant

Kyoto encyclopedia of genes and genomes (KEGG) analysis was performed to better clarify the signaling pathways associated with the DEGs in response to CYVCV infection. The DEGs with a KO ID could be classified into pathway items. The significantly enriched pathways for up- and down-regulated DEGs (*p*-value ≤ 0.05) are displayed in Fig. 6 and Table S4. These included ‘DNA replication’, ‘ribosome’, ‘monoterpenoid biosynthesis’, ‘mismatch repair’, ‘phenylpropanoid biosynthesis’, ‘homologous recombination’, ‘flavonoid biosynthesis’, ‘alpha-Linolenic acid metabolism’, ‘linoleic acid metabolism’, and ‘pyrimidine metabolism’ for up-regulated DEGs; while ‘photosynthesis’, ‘pentose and glucuronate interconversions’, ‘brassinosteroid biosynthesis’, ‘zeatin biosynthesis’, ‘stilbenoid, diarylheptanoid and gingerol biosynthesis’, ‘nitrogen metabolism’, ‘ABC transporters’, ‘limonene and pinene degradation’, ‘diterpenoid biosynthesis’, ‘Fructose and mannose metabolism’ and ‘thiamine metabolism’, and ‘carbon fixation in photosynthetic organisms’ for down-regulated DEGs (Fig. 6; Table S4).

### Plant hormones-related genes response to CYVCV infection

**Fig. 7:**
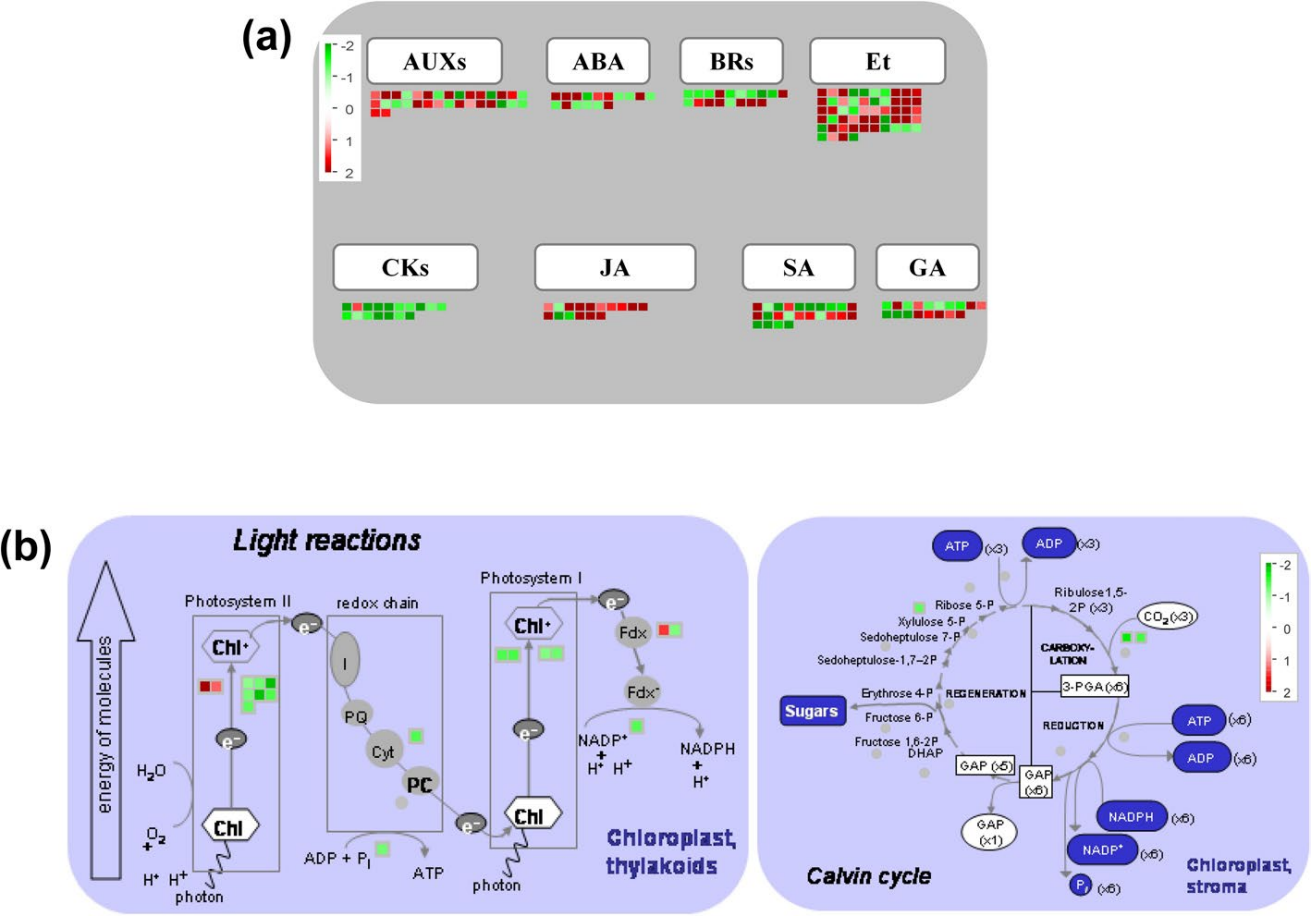
Mapman views of phytohormone-related genes (**a**) and photosynthesis-related genes (**b**) that were regulated in lemon plants after CYVCV infection [76]. Genes significantly up-regulated and down-regulated by CYVCV infection are displayed in red and green, respectively. ABA, abscisic acid; AUXs, auxins; BRs, brassinosteroids; CKs, cytokinins; Et, ethylene; GA, gibberellin; JA, jasmonic acid; SA, salicylic acid

To explore the role of phytohormone in lemon plants in response to CYVCV infection, Mapman categorized DEGs into the ‘phytohormone-regulation’ to analyze the gene expression expression patterns of pathways related to AUXs, ABA, BRs, Et, CKs, JA, SA, and GA (Fig. 7). As shown in Fig. 7 and Table S5, these phytohormone-related genes displayed different responses towards CYVCV infection. A total of 32 genes associated with the biosynthesis and signal transduction of AUXs were significantly regulated by CYVCV infection, and the majority of these DEGs were up-regulated. Among them, 15 of the 23 DEGs involved in the ‘auxin induced-regulated-responsive-activated’ were up-regulated, and 4 of the 5 DEGs associated with the ‘auxin synthesis’ were up-regulated. Moreover, half of the DEGs related to the biosynthesis and signal transduction of ABA were up-regulated, and the other half were down-regulated. Among them, 5 of the 6 DEGs involved in the ‘abscisic acid synthesis’ were up-regulated, while 5 of the 8 DEGs associated with ‘abscisic acid induced-regulated-responsive-activated’ were down-regulated. Almost all the ‘brassinosteroid synthesis’-related DEGs were down-regulated, while most of the DEGs involved in ‘ethylene synthesis’ and ‘ethylene signal transduction’ were up-regulated. Additionally, almost all of the DEGs associated with ‘cytokinin synthesis’ and ‘cytokinin signal transduction’ were down-regulated, while most of the DEGs involved in ‘jasmonate synthesis’ and ‘jasmonate signal transduction’ were up-regulated. Furthermore, most of the DEGs associated with ‘salicylic acid synthesis’ were down-regulated. In the pathways of GA metabolism, half of the DEGs were up-regulated, and the other half were down-regulated.

**Fig. 8:**
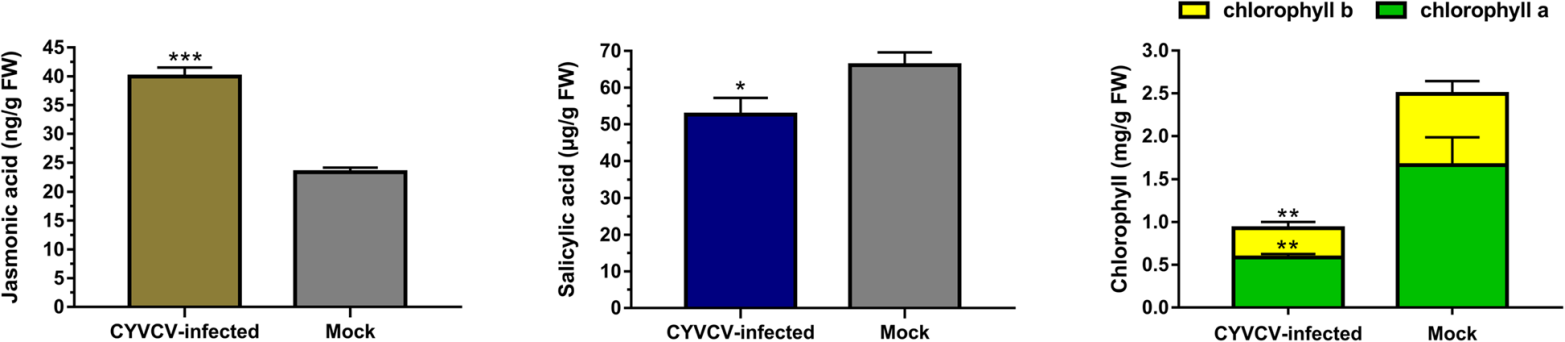
The jasmonic acid, salicylic acid and chlorophyll contents in lemon leaves. Mock, the mock-inoculated lemon plants; CYVCV-infected, the CYVCV-infected lemon plants. Statistically significant differences of CYVCV-infected plants versus mock at *p*-values ≤ 0.001 (***), *p*-values ≤ 0.01 (**) and ≤ 0.05 (*) are indicated

In addition, the one-step double-antibody sandwich enzyme-linked immunosorbent assay (ELISA) revealed that JA content increased significantly in the early stage of CYVCV-infection, while SA content decreased significantly (Fig. 8). These results showed that changes in hormone levels were consistent with transcriptome results.

### Photosynthesis-related genes response to CYVCV infection

Previous studies have revealed that virus infection can modify photosynthesis, thus disrupting chloroplast components and function. To assess the effects of CYVCV infection on the photosystem, the expression patterns of photosynthesis-related DEGs were analyzed by Mapman. As shown in Fig. 7 and Table S6, almost all of the photosynthesis-related DEGs were down-regulated. Six genes, encoding photosystem II (PSII) reaction center subunit A, C, D and T (PsbA, PsbC, PsbD and PsbT) as well as PSII oxygen evolving enhancer protein 2 and 3 (PsbP and PsbQ), which are components of PSII, were down-regulated. Similarly, four genes, encoding photosystem I (PSI) chlorophyll a-b binding protein 4 and 7, as well as PSI P700 apoprotein A1 and A2 (PsaA and PsaB), which are components of PSI, were also down-regulated. Moreover, three genes, encoding ferredoxin-NADP reductase (FENR1), a novel subunit of the chloroplast NAD(P)H dehydrogenase complex (PNSB3), and the cytochrome b(6) subunit of the cytochrome b6f complex (Cyb6), which are associated with cyclic electron flow around photosystem, were down-regulated. Furthermore, three genes, encoding ribulose-phosphate 3-epimerase (RPE) and ribulose 1,5-bisphosphate carboxylase/oxygenase large and small subunits (RbcL and RbcS), which are involved in Calvin cycle, were down-regulated.

**Fig. 9:**
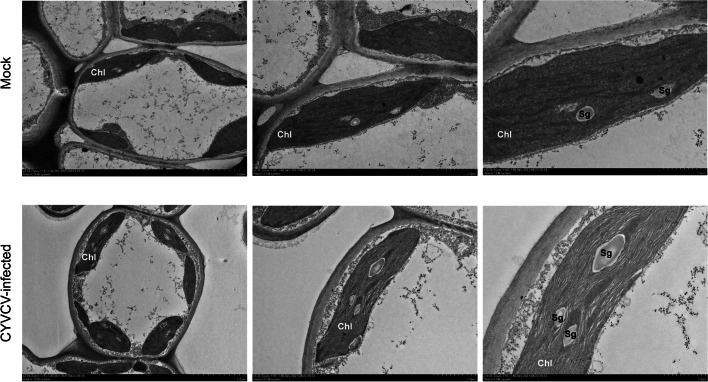
Electron microscopic images of the chloroplasts of palisade tissue in the lemon leaves. Mock, the mock-inoculated lemon plants; CYVCV-infected, the CYVCV-infected lemon plants; Chl, chloroplast; Sg, starch granule

Furthermore, to determine the effects of CYVCV infection on chloroplast, comparable observations of chloroplast structure between mock and CYVCV-infected lemon leaves were conducted by transmission electron microscopy. In mock plants, the chloroplasts had a complete external envelope and clear boundary, the lamella structure pile folds were in order, the thylakoids were well-developed with good shape, and both stroma and grana lamellae were arranged in a compact manner **(**Fig. 9**)**. However, the chloroplasts of CYVCV-infected leaves were less dense, with loosed grana lamellae of thylakoid and fewer thylakoid stacks of grana lamellae than those of mock plants, and the stacks had an irregular shape and were unevenly distributed. Moreover, the micro-graphs indicated that the chloroplasts of CYVCV-infected leaves contained more starch grains compared to those of mock leaves (Fig. 9). Additionally, The chlorophyll content assay showed that the contents of chlorophyll a and chlorophyll b in CYVCV-infected lemon were significantly lower compared with mock treated plants (Fig. 8).

### RT-qPCR verification of transcriptomics data

**Fig. 10:**
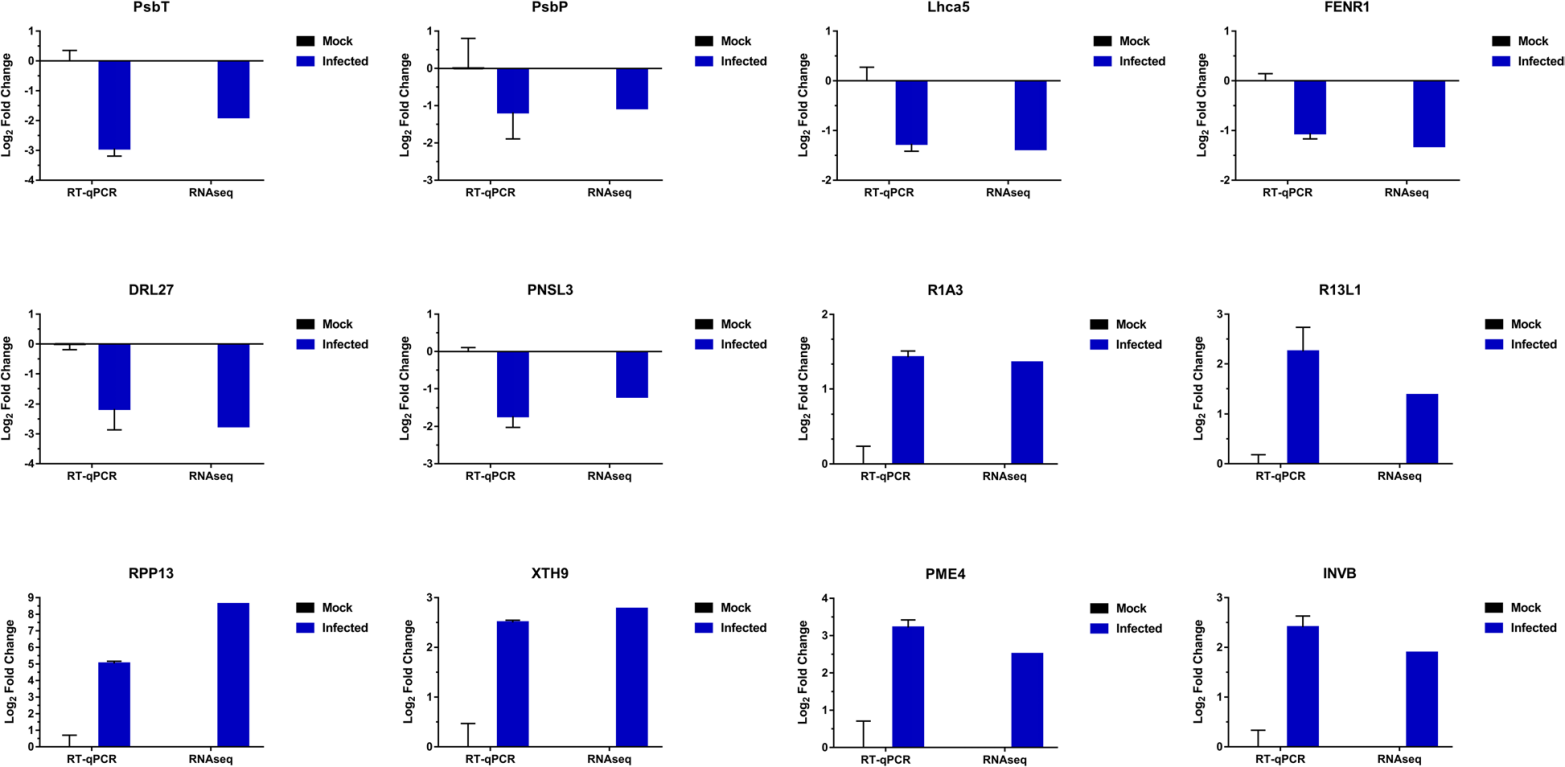
Comparison of the relative expression levels of 12 selected genes detected by RNAseq and RT-qPCR. All RT-qPCR reactions included 3 biological replicates and repeated for 3 times. The values are shown as mean ± SEM. Relative expression levels were determined the using 2^–ΔΔCt^ method and log_2_ normalized. Actin was the reference gene used for RT-qPCR assays

To verity the RNA-seq data, 12 DEGs were chosen for RT-qPCR assays, including 6 down-regulated DEGs, *PsbP*, *PsbT*, *Lhca5* (photosystem I light harvesting complex gene 5), *FENR1* (ferredoxin-NADP reductase), *DRL27* (disease resistance protein At4g27190-like isoform X1) and *PNSL3* (photosynthetic NDH subunit of lumenal location 3), as well as 6 up-regulated genes, *R1A3* (synaptic vesicle membrane protein VAT-1 homolog isoform X2), *R13L1* (NBS-LRR type disease resistance protein), *RPP13* (disease resistance protein RPP13), *XTH9* (xyloglucan endotransglucosylase/hydrolase protein 9-like), *PME4* (putative thermostable pectinesterase) and *INVB* (soluble acid invertase). The results indicated that the expression patterns of all selected genes verified by RT-qPCR were in good agreement with those detected by transcriptome sequencing (Fig. 10).

## Discussion

Recently, high-throughput RNA-seq has been widely applied to evaluate virus-plant interaction. In the present study, RNA-seq was used to examine the transcriptomics profiles of lemon after CYVCV infection and identify DEGs between mock- and CYVCV-infected lemon plants. To our knowledge, this study is the first large-scale transcriptome analysis of lemon plants infected with CYVCV. The results demonstrated the significant changes in photosynthesis and phytohormone metabolism and signaling pathways in lemon plants after CYVCV infection.

### Roles of phytohormone in regulating lemon-CYVCV interactions

Phytohormone are tuners of plant responses to abiotic and biotic stresses. They are involved in a variety of complex networks, through which they modulate responses to different stimuli [24]. The successful establishment of systemic viral infection in host plants is usually associated with changes in phytohormone metabolism and signaling, leading to hormonal disruption, which is characterized by the simultaneous activation of several antagonistic phytohormones [12, 25, 26]. Thus, we analyzed the response of lemon plants to CYVCV based on different phytohormone biosynthesis and signal transduction pathways.

### AUXs-regulated response

AUXs play an important role in plant growth and development by sustaining apical dominance, and the Aux signaling/responsive factor mutants show abnormal growth phenotypes (Benjamins and Scheres, 2008). Many viruses can lead to aberrant phenotypes, such as loss of apical dominance, leaf curling and stunted growth, resembling those of mutants with compromised AUXs biosynthesis and/or signaling (Kazan and Manners, 2009). Moreover, AUXs are found to antagonize SA pathway, which often play a negative role in plant defense against viruses by increasing plant susceptibility and viral systemic movement [12]. A subset of AUX response elements is crucial for the replication and transport of some viruses, including Tobacco mosaic virus (TMV) [27, 28]. In this study, the expression levels of genes encoding auxin-responsive proteins SAUR72, SAU41, B561P, B561J, and ARG7 were up-regulated after CYVCV infection. SAUR72 and SAU41 belong to SAUR family, which play a pivotal role in regulating plant growth and development [29, 30]. Thus, we speculate that CYVCV disrupts the lemon plants’ auxin response system to reprogram the cellular environment into a more compatible one for virus replication and spread.

### CKs-regulated response

CKs are primarily generated in the meristemic zone of shoots and subsequently translocated to actively growing regions. They not only induce cell proliferation and elongation, but also delay leaf senescence and regulate various signaling cascades [31, 32]. Additionally, CKs exhibit synergistic effects on SA signaling pathway, and plant-derived CKs trigger defense responses to biotrophs in a SA-regulated manner [33]. For white clover mosaic virus (WClMV)-infected Phaseolus vulgaris, WClMV infection has been shown to reduce the active forms of CK within the first day of infection. Treatment with 50 nM/L dihydrozeatin (a cytotaxin) can reduce the viral RNA and CP level of WClMV [34, 35]. In this study, the decreased expression of many DEGs involved in cytotaxin biosynthesis and signal transduction, including IPT5 (adenylate isopentenyltransferase 5) and AHK5 (histidine kinase 5) was detected, suggesting that CYVCV infection can inhibit the biosynthesis and signaling pathway of CKs in lemon plants, and possibly in turn lead to an abnormal leaf growth.

### Et-regulated response

Et is involved in the defense response to necrotrophic plant pathogens and cellular senescence. [36]. Et seems not to be involved in plant defense against viruses, and as AUXs, it also antagonizes the pathways downstream of SA signaling [12]. Et is associated with symptom formation after cauliflower mosaic virus (CaMV) infection, systemic movement of TMV crucifier-infecting strain (TMVcg), and formation of necrotic lesions [37, 38]. The *ACS1* (1-aminocyclopropane-1-carboxylate synthase 1) mutant in Et pathway is resistant to TMVcg. Moreover, 1-aminocyclopropane-1-carboxylic acid (ACC) can enhance the accumulation of TMVcg in treated plants. The study also showed that *ACS6* (1-aminocyclopro-pane-1-carboxylate synthase 6) was remarkably up-regulated in *wrky8* mutants. WRKY transcription factors have been found to induce different defense mechanisms, and play an essential role in modulating plant defense genes [37]. Similarly, in this study, the expression of DEGs involved in ethylene synthesis, (e.g. *ACS1*, *ACS6*, *ACCH1* (1-aminocyclopropane-1-carboxylate oxidase homolog 1) and *ACCH6* (1-aminocyclopropane-1-carboxylate oxidase homolog 6)) were up-regulated by CYVCV, suggesting that Et may promote the systemic movement and viral accumulation of CYVCV in lemon plants.

### JA-regulated response

JA belongs to a large family of oxygenated fatty acids (oxylipins) that are resistant to insect infestation and infection by necrotrophic pathogens [39]. JA has both negative and positive effects on plant defense against viruses, and appears to facilitate plant defenses at early stages of infection. However, it can decrease plant resistance if it occurs at later stages [40, 41]. JA and SA have antagonistic or synergistic interrelations, which modulate defense pathways and display antagonistic interaction [42]. For instance, treatment with JA at the initial stage of potato virus Y (PVY) and potato virus Y (PVX) double infections improved plant resistance, but later application induced vulnerability, which might be attributed to the antagonistic effect of JA on SA [40]. A previous study showed that knockdown of the JA biosynthesis gene *AOS* (allene oxide synthase) could enhance plant resistance, while exogenous application of methyl jasmonate (MeJA) could reduce local resistance to TMV and permit systemic movement, suggesting that MeJA abolishes plant defense against TMV. The study also indicated that the improved resistance of AOS-silenced plants was part of the elevated level of SA in the plants [43]. *AOCs* (allene oxide cyclasees), which are also key genes for the synthesis of JA and MeJA, including *AOC3* and *AOC4*, were up-regulated by CYVCV in this study. Moreover, JA content of lemon plant increased significantly in the early stage of CYVCV-infection, while SA content decreased significantly. Therefore, we speculate that JA synthesis is induced in lemon plants at the initial stage of CYVCV infection to enhance plant resistance, but as JA antagonizes the SA pathway, the continuous up-regulation of JA synthesis enhances plant sensitivity and contributes to viral systemic infection.

### Other phytohormones-regulated responses

Other phytohormones, such as SA, GA, ABA and BRs, are also involved in plant growth and development, and regulate plant defense to pathogens. SA is one of the phenolic compounds produced by plants after pathogenic infection, which is essential for inducing both local and systemic resistance [44, 45]. The significance of SA arises from its regulatory effects on resistance (R)-gene and basal immune responses, and based on the positive association between SA-regulated plant defense and siRNA antiviral machinery [46–48]. In this study, SA pathway may be antagonized by JA, AUXs and Et. At the same time, multiple genes responsible for SA biosynthesis were significantly down-regulated by CYVCV infection, resulting in the inhibition of SA-mediated plant resistance. ABA enhances callose accumulation on plasmodesmata and inhibits cell-to-cell transmission of viruses, including tobacco necrosis virus and TMV. Moreover, it antagonizes SA signaling and attenuates resistance at local infection sites by suppressing HR activation, reducing the levels of SA and ROS, and weakening distal systemic acquired resistance and siRNA systems [42, 49]. GA facilitates plant defense to necrotrophs or biotrophs by maintaining the balance between SA and JA/Et signaling pathways [50]. Furthermore, BR-stimulated plant defense to biotrophs appears to be independent of SA [51]. In our study, many genes involved in ABA biosynthesis were remarkably up-regulated in lemon plants after infection with CYCVV, while most genes involved in ABA signal transduction and response were down-regulated, suggesting that ABA has multiple functions and may regulate the response of lemon plants to CYVCV infection. Conversely, many genes associated with the biosynthesis of GA and BRs were markedly down-regulated in lemon plants after infection with CYCVV, while most of the genes were involved in GA and BR signal transduction and response, indicating that GA and BR pathways were activated in CYVCV-infected plants.

### Disordered photosynthesis is an important factor involved in symptom development

Leaf chlorosis is one of the most common symptoms associated with virus infection, which is characterized by chloroplast structural changes and altered pigmentation. The influences of viruses on chloroplast structure and function often lead to a decline in photosynthetic activity. A growing number of studies on a wide range of virus-plant interactions have indicated that viral infection can inhibit host photosynthesis [18, 19, 52–54]. Previous studies have shown that inhibition of photosynthesis is an important strategy for pathogenesis employed by viruses to facilitate infection [55–57]. The disruption of normal chloroplast constituents and functions can also affect the development of chlorosis symptoms related to viral infection [23].

In this study, almost all DEGs involved in each pathway (PSII, PSI, electron carrier and ATP synthase of light reaction, and Calvin cycle) of photosynthesis were down-regulated by CYVCV infection. Moreover, almost all of the proteins encoded by these genes are required for photosystem assembly and thylakoid membrane stabilization. The PsbP protein is one of the components of the PSII oxygen-evolving complex (OEC) and the thylakoid lumenal subunit of PSII, which is necessary for the assembly and stabilization of PS II [58–60]. Previous studies have shown that in PsbP-RNAi leaves, another component of OEC, the PsbQ protein, cannot bind to the thylakoid membrane, and is then degraded by proteases in the thylakoid lumen. Meanwhile, for PSbP-RNAi leaves, light harvesting complex of photosystem II (LHCII) dissociates from the PSII core dimer, resulting in the failure of PSII to assemble normally and a remarkable decrease in PSII activity, which in turn leads to the changes in thylakoid membrane structure, blurred thylakoid lamellae, and disordered grana stacking, with a chlorotic phenotype [61–63]. Furthermore, it has been previously shown that silencing of *RbCS* (Rubisco small subunit) could induce leaf chlorosis in *N. benthamiana* and enable tomato mosaic virus (ToMV) to promote necrosis in the inoculated leaves, thereby leading to an increase in viral infectivity. This implies that *RbCS* plays an important role in plant antiviral defenses [64]. Additionally, chlorophyll biosynthesis is related to the formation of thylakoid membranes [65, 66]. In this study, the chloroplasts of CYVCV-infected lemon plants showed less dense thylakoid lamellae, a looser thylakoid structure and less severe grana stacking compared to those of mock-inoculated lemon plants. Furthermore, after CYVCV-infection, the chlorophyll content of the lemon leaves decreased significantly. Therefore, we speculate that CYVCV infection downregulates the expression levels of PsbP, PsbQ, and RbCS in lemon plants, thus causing damage to the thylakoid membrane structure of chloroplasts, blocking chlorophyll synthesis, and resulting in yellow and clearing vein in the infected leaves. At the same time, due to the inhibition of photosynthesis, it further aggravates CYVCV infection in lemon plants.

## Conclusions

This study indicates that CYVCV infection remarkably impacts the physiological characteristics of lemon plants, including photosynthetic capacity and phytohormone metabolism and signaling. The metabolic and signaling pathways associated with photosynthesis and phytohormones in lemon plants are involved in symptom development after CYVCV infection. More notably, CYVCV infection has regulatory effects on the biosynthesis and signaling of AUXs, CKs, JA and ET, as well as the inhibition of SA signaling pathway, which possibly in turn lead to an enhanced systemic infection of CYVCV. Additionally, the inhibition of photosynthesis pathway probably contribute to yellowing leaf symptom in CYVCV-infected lemon plants. Compilation of genes associated with photosynthesis and phytohormone pathways can provide an ideal candidate gene list to evaluate the interaction between CYVCV and citrus plants. Our findings offer new insights into the molecular basis of symptom development in lemon plants after CYVCV infection.

## Materials and methods

### Virus, plant materials, and inoculation process

The two-year-old disease-free ‘Eureka’ lemon plants were obtained and licensed from from the virus-free citrus repository of the National Citrus Virus Exclusion Center (NCVEC) in Southwest University were used for inoculation via grafting. Each ‘Eureka’ lemon plant was grafted with four buds. The buds used for CYVCV-inoculation were from ‘DaiDai’ sour orange (*C. aurantium* L.) solely infected with CYVCV maintained in a greenhouse in NCVEC. CYVCV-free buds from disease-free ‘Daidai’ sour orange were used as the mock inoculation. The inoculated plants were maintained in a greenhouse at 23 °C under a 16 h photo-period.

### RNA construction for RNA-seq

The fully-expanded top leaves were collected from mock-inoculated and CYVCV-inoculated plants every fifteen days for RNA isolation with Trizol reagent (Invitrogen, USA) according to the manufacturer’s instructions until the virus was detected. Systemic viral infection was then assessed by RT-PCR assays as previously described [67]. Total RNA samples of three independent CYVCV-infected plants in the initial stage of infection (named as Infected-1, Infected-2, and Infected-3) and mock-inoculated plants at the same time after grafting (named as Mock-1, Mock-2, and Mock-3) were prepared. To avoid random sampling bias within a single sample, the total RNA of infected-1, infected-2, infected-3, Mock-1, Mock-2, and Mock-3 were equally pooled for further analysis. The RNA samples of mock and CYVCV-infected plants were subjected to library construction. RNA-seq was performed using the Illumina HiSeq™ 2000 platform at Kidio Biotechnology Co., Ltd. (Guangzhou, China).

### Data filtering and mapping

To obtain clean reads, the raw reads containing adapter sequences and low-quality sequences (the rate of reads with quality value ≤ 20 is more than 50% or uncertain base ‘N’ is more than 10% were eliminated. Currently, no published genomic databases report for *C. limon*, thus the clean reads were then mapped to the reference genome of congeneric species, *C. sinensis* (http://www.ncbi.nlm.nih.gov/genome/10702) [68] by TopHat2 [69] using the parameters set by the system. To reduce any sequence redundancy, the contigs were further connected after the paired-end reads, and sequences that could not be extended on either end were defined as unigenes.

### Gene-level identification and quantification of DEGs

The FPKM values of all genes were employed to determine the expression levels of unigenes by using cufflinks (version 2.2.1) [70, 71]. Additionally, the method of DESeq [72] was used to screen DEGs between of mock and CYVCV-infected plants. A two-fold change (< − 1 or > 1 in log_2_ ratio value) and FDR ˂ 0.05 were set as the threshold for DEGs. Hierarchical cluster analysis (HCA) was conducted to determine the expression pattern of each DEG.

### Functional and pathway analysis of DEGs

To explore the molecular functions of DEGs, GO enrichment analysis was conducted after mapping all DEGs to GO terms in the database (http://www.geneontology.org) by a Fisher’s exact test with Bonferroni correction. An enriched term with *p*-value < 0.01 was deemed statistically significant. Meanwhile, KEGG [73–75] was conducted to explore the pathways associated with DEGs. The significantly enriched pathways were screened using the Fisher’s exact test based on hypergeometric distribution. An enriched pathway *p*-value < 0.05 was deemed statistically significant. Subsequently, the MapMan ontologies of Citrus sinensis were imported into the MapMan tool. To this end, all DEGs in this study were mapped to MapMan bins (version 3.5.1) for data visualization and specific pathway analysis [76].

### Electron microscopic analysis

Small-size tissues (4 mm × 1 mm) were excised from the leaves of mock and CYVCV-infected lemon plants. The sampled tissues were fixed for 2 h in 2.5% glutaraldehyde (pH 7.4). After washing 3 times with 0.1 M phosphate buffer (pH 7.2), the tissues were fixed again for 2 h in 1% osmic acid. Subsequently, the tissues were dehydrated in a graded series of ethanol, embedded in Epon-Araldite resin for penetration, and placed in a mold for polymerization. The ultrathin sections were prepared and subjected to microstructural analysis. After counterstaining with 2.7% lead citrate and 3% uranyl acetate, the sections were evaluated using a HT7800 transmission electron microscope.

### Real time-quantitative PCR (RT-qPCR) validation

SYBR Green-based RT-qPCR was performed to detect the expression level of each selected gene. The RNA extracts (1 μg) of mock and CYVCV-infected plants were used for cDNA synthesis using a PrimeScriptTM RT Reagent Kit with a gDNA Eraser (TaKaRa, Dalian, China) by following the kit’s protocol. RT-qPCR was carried out using the NovoStart SYBR qPCR SuperMix (Novoprotein, Shanghai, China) on the qTOWER3 Real-Time System (Analytik Jena, Jena, German). The RT-qPCR cycles consisted of an initial denaturation step of 30 s at 94 °C, followed by 40 cycles of 5 s at 95 °C and 34 s at 60 °C. The citrus gene *Actin* was amplified as a standard reference for normalizing the expression level of each gene. The primer sequences (Table S1**)** of target and reference genes were designed based on the unigene sequences and citrus sequences from GenBank. All reactions were conducted with three biological and technical replicates. The 2^−ΔΔCT^ method [77] was utilized to measure the relative expression level of each gene.

### Measurement of SA, JA and Chlorophyll

The SA and JA contents in mock and CYVCV-infected lemon leaves were determined with the Plant SA ELISA kit (#YX-001901P) and Plant JA ELISA kit (#YX-001001P) (Sinobestbio, Shanghai, China), respectively. Chlorophyll content of the leaves of mock and CYVCV-infected lemon plants was measured using the alcohol and acetone extraction, spectrophotometer colorimetric method according to the kit instructions (#YX-W-700) (Sinobestbio, Shanghai, China).

## Supplementary Information

**Additional file 1:** **Table S1.** Primers for RT-qPCR.

**Additional file 2:** **Table S2.** Differentially expressed genes (DEGs) in CYVCV-infected and mock plants.

**Additional file 3:** **Table S3.** GO enrichment analysis of DEGs.

**Additional file 4:** **Table S4.** KEGG pathway analysis of DEGs.

**Additional file 5:** **Table S5.** Differential expression of phytohormone-related genes in plants after CYVCV infection.

**Additional file 6:** **Table S6.** Differential expression of photosynthesis-related genes in plants after CYVCV infection.

## Data Availability

The raw reads of our RNA-seq and DGE data were deposited in the Sequence Read Archive under accession numbers SRR19214145, SRR19214146, SRR19214147, SRR19214148, SRR19214149, SRR19214150.

## Abbreviations

CYVCV: *Citrus yellow vein clearing virus*
DEGs: Differentially expressed genes
GO: Gene ontology
KEGG: Kyoto encyclopedia of genes and genomes
