# Supplementary material for: Transcriptome analysis of Citrus limon infected with Citrus yellow vein clearing virus
Source: BMC Genomics. 2023 Feb 7;24:65. doi: 10.1186/s12864-023-09151-5 (PMC9903606; doi:10.1186/s12864-023-09151-5)
Supplement: Supplementary file 1 — Additional file 1: Table S1. Primers for RT-qPCR. [file 12864_2023_9151_MOESM1_ESM.doc]

**Table S1. Primers for RT-qPCR**

| Primer name | Primer sequence(5′-3′) | Symbol | mRNA ID |
| --- | --- | --- | --- |
| PsbT-F | TGAATGTTGGGGCGAGCAAT | PsbT | XM_006482801.3 |
| PsbT-R | TTTCTTGGCTTCTGGGGTGC |
| PsbP-F | CGAATGAAGACCTCCGTGTT | PsbP | XM_006473790.3 |
| PsbP-R | CCACACTGACGCTTGAACTA |
| Lhca5-F | ATCAGAAATGCTCGCGACTG | Lhca5 | XM_006473266.3 |
| Lhca5-R | ATAGCAAATGGCCTTGCAATTTAT |
| FENR1-F | GGCATTGATTGGCTGGACTA | FENR1 | XM_006466890.3 |
| FENR1-R | TAGCCGTAAATTGCTCCGTT |
| DRL27-F | GGGAACTGTCAAAAGGAGCA | DRL27 | XM_006471087.3 |
| DRL27-R | GCTGTAACTTGGTCGCCATA |
| PNSL3-F | GGAACCAAAGGCAGGATTCA | PNSL3 | XM_006475236.3 |
| PNSL3-R | GTTGCTTATCAGTGGGTGCT |
| R1A3-F | AGAGATACTGTCTGTGGGGC | R1A3 | XM_006490563.3 |
| R1A3-R | TAGGATCTTCCTCTGCGCTT |
| R13L1-F | TCCGAAGGATACCCACAACT | R13L1 | XM_006482873.3 |
| R13L1-R | TGCCGACTAAGGGTATCACA |
| RPP13-F | TGGAGTGGCAAGTAGAGGAA | RPP13 | XM_006470582.3 |
| RPP13-R | AAGAATCTGCCACCGAAGAG |
| XTH9-F | GCCAGTGTCTAGTGAGGTCT | XTH9 | XM_006469681.3 |
| XTH9-R | TGAAGACAGAAGAACGGGGA |
| PME4-F | CCCCAATCCAAACCAAAGGA | PME4 | NM_001288917.1 |
| PME4-R | CTTCCATGGCCTGCCTAAAT |
| INVB-F | ACCAAACGACCGACTTCAAA | INVB | NM_001288945.1 |
| INVB-R | GGTCCCGATTGCATAATGGT |
